# Vascular endothelial growth factor C promotes cervical cancer metastasis via up-regulation and activation of RhoA/ROCK-2/moesin cascade

**DOI:** 10.1186/1471-2407-10-170

**Published:** 2010-04-29

**Authors:** Mian He, Yang Cheng, Wen Li, Qiongshan Liu, Junxiu Liu, Jinghe Huang, Xiaodong Fu

**Affiliations:** 1 Department of Gynecology and obstetrics, the first affiliated hospital of Sun Yat-sen University, Guangdong, Guangzhou, 510089, China; 2 Department of Gynecology and obstetrics, Guangzhou first municipal people's hospital, Guangdong, Guangzhou, 510180, China; 3 Laboratory of General Surgery, the first affiliated hospital of Sun Yat-sen University, Guangdong, Guangzhou, 510089, China; 4 Department of Physiology, Zhongshan School of Medicine, Sun Yat-sen University, Guangdong, Guangzhou, 510089, China

## Abstract

**Background:**

The elevated expression of vascular endothelial growth factor C (VEGF-C) is correlated with clinical cervical cancer metastasis and patient survival, which is interpreted by VEGF-C functions to stimulate angiogenesis and lymphatic genesis. However, the direct impact of VEGF-C on cervical cancer cell motility remains largely unknown.

**Methods:**

In this study, we investigated the effects of VEGF-C on actin cytoskeleton remodeling and on cervical cancer cell migration and invasion and how the actin-regulatory protein, moesin regulated these effects through RhoA/ROCK-2 signaling pathway.

**Results:**

On cervical carcinoma cell line SiHa cells, exposure of VEGF-C triggered remodeling of the actin cytoskeleton and the formation of membrane ruffles, which was required for cell movement. VEGF-C significantly enhanced SiHa cells horizontal migration and three-dimensional invasion into matrices. These actions were dependent on increased expression and phosphorylation of the actin-regulatory protein moesin and specific moesin siRNA severely impaired VEGF-C stimulated-cell migration. The extracellular small GTPase RhoA/ROCK-2 cascade mediated the increased moesin expression and phosphorylation, which was discovered by the use of Y-27632, a specific inhibitor of Rho kinase and by transfected constitutively active, dominant-negative RhoA as well as ROCK-2 SiRNA. Furthermore, in the surgical cervical specimen from the patients with FIGO stage at cervical intra-epithelial neoplasia and I-II cervical squamous cell carcinoma, the expression levels of moesin were found to be significantly correlated with tumor malignancy and metastasis.

**Conclusions:**

These results implied that VEGF-C promoted cervical cancer metastasis by upregulation and activation of moesin protein through RhoA/ROCK-2 pathway. Our findings offer new insight into the role of VEGF-C on cervical cancer progression and may provide potential targets for cervical cancer therapy.

## Background

Cervical cancer is the second most common cancer in women worldwide and it has been steadily increasing in young women [1]. Human papillomavirus (HPV) infection causes virtually all cases of cervical cancer. Recent years the great advancement of prophylactic vaccines which aim directly at different types of HPV has been achieved and results in a substantial reduction in the incidence of cervical cancer [2]. Notwithstanding, many issues related to the radical therapy of existing cervical cancer remain unresolved. One major problem is the cervical cancer metastasis, which is the leading cause of mortality.

Cancer metastasis is composed of a complex series of phenotypic and biochemical changes such as angiogenesis, lymphangiogenesis, gene expression, motility or cell shape. These alterations are regulated by several sets of growth factors and their cognate receptors [3]. Vascular endothelial growth factor C (VEGF-C), a dimeric glycoprotein belonging to VEGF family of cytokines, is found to be implicated in the most aggressive tumors. By binding to its receptor Flt-4, VEGF-C promotes angiogenesis and/or lymphangiogenesis, thus accelerates cancer metastasis to lymph nodes and distant organs [4,5]. Correspondingly, clinical studies have verified that VEGF-C expression is closely related to invasion phenotype and affects the patient's survival in cervical carcinomas [6-8]. Previously, it was reported that the Flt-4 expression was restricted in the endothelial cells of lymphatic vessels. Recent years the Flt-4 has been shown to be expressed in a variety of human malignancies, including cervical cancer [9,10]. These observations hint the possibility that as the specific ligand to Flt-4, VEGF-C may be implicated in cervical cancer progression by direct impacts on tumor cells.

Cell migration is critical to cancer cell invasion and metastasis. The first step is represented by dynamic filamentous actin cytoskeletal remodeling, which allows the formation of protrusions that adhere to the extra-cellular matrix and generate intra-cellular forces for cell movement [11,12]. Indeed, actin remodeling is involved in cancer transformation and metastasis [13]. Our previous work indicated that actin remodeling is the primary step, not only for cancer cell metastasis [14], but also for endothelial and neuron cell migration [15,16]. These events are mediated by moesin, an actin-binding protein belonging to the ezrin/radixin/moesin (ERM) family. Indeed, moesin gene expression is shown to be strongly associated with metastatic phenotypes of cervical cancer [17]. There is also evidence showing that VEGF-C enhances cervical cancer cell motility [10], but the underlying mechanisms remain largely unknown. In the present study, we purpose to investigate the effects of VEGF-C on actin cytoskeleton remodeling and on cervical cancer cell migration and to characterize the role of moesin and the signaling cascade implicated in these actions.

## Methods

### Cell cultures and treatments

An established cervical carcinoma cell line (SiHa) was used for this study. SiHa cells were incubated in RPMI 1640 medium containing 10% fetal calf serum (FCS), L-glutamine and penicillin streptomycin under a 5% CO_2 _atmosphere at 37°C. An inhibitor was always to be added 1 h before starting the treatments. Recombinant human VEGF-C wild type (2179-VC-025) and the fusion protein of human IgG with the extracellular ligand-binding domains of Flt-4 (Flt-4/IgG) (349-F4-050) were purchased from R&D Systems (Minneapolis, MN). The selective inhibitor of the Rho-associated protein kinase Y-27632 was from Sigma-Aldrich (Saint-Louis, MO).

### Immunoblottings

Cell lysates were separated by SDS-PAGE. Antibodies used were: moesin (clone 38, Transduction Laboratories, Lexington, KY), Thr^558^-P-moesin (sc-12895, Santa Cruz Biotechnology, Santa Cruz, CA), RhoA (sc-418, Santa Cruz), ROCK-2 (sc-5561, Santa Cruz), β-actin (sc-1615, Santa Cruz). Primary and secondary Abs were incubated with the membranes with standard technique. Immunodetection was accomplished using enhanced chemiluminescence. Chemiluminescence was acquired with a quantitative digital imaging system (Quantity One, BioRad, Hercules, CA) allowing to check for saturation. Densitometry values were adjusted to β-actin intensity and normalized to expression level from the control sample.

### Kinase assays

SiHa cells were harvested in 20 mM Tris-HCl, 10 mM EDTA, 100 mM NaCl, 0.5% IGEPAL and 0.1 mg/mL PMSF. Equal amounts of cell lysates were immunoprecipitated with 40 μg Rhotekin RBD agarose (14-383, upstate) vs. GTP-RhoA or an Ab vs. ROCK-2 (sc-5561, Santa Cruz). The IPs were washed three times with buffer containing 20 mM Tris-HCl, 10 mM EDTA, 150 mM NaCl, 0.1% IGEPAL and 0.1 mg/mL PMSF. For ROCK-2 activity assay, two additional washes were performed in kinase assay buffer (20 mM MOPS, 25 mM b-glycerophosphate, 5 mM EGTA, 1 mM DTT) and the samples were therefore resuspended in this buffer. 5 mg of de-phosphorylated myelin basic protein (Upstate) together with 500 mM ATP and 75 mM MgCl_2 _were added to each sample and the reaction was started at 30°C for 20 min. The reaction was stopped on ice and the samples were resuspended in Laemmli Buffer. The samples were separated with SDS-PAGE and Western blot analysis was performed using antibodies recognizing RhoA (sc-418, Santa Cruz) or Thr^98^-P-myelin basic protein (05-429, Upstate).

### Cell immunofluorescence

SiHa cervical cancer cells were grown on coverslips and exposed to treatments. Cells were fixed with 4% paraformaldehyde for 30 min and permeabilized with 0.1% Triton X for 5 min. Blocking was performed with 3% normal serum for 20 min. After washing the nuclei were counterstained with 4'-6-diamidino-2-phenylindole (DAPI) (Sigma) and actin was stained with Texas Red-phalloidin (Sigma). The coverslips were mounted with Vectashield mounting medium (Vector Laboratories, Burlingame, CA). Immunofluorescence was observed by an Olympus BX41 microscope and images were captured by DP70 Olympus digital camera with high-resolution.

### Transfection experiments

Each plasmid (15 μg) was transfected into SiHa cervical cancer cells using the Lipofectamine (Invitrogen) according to the manufacturer's instructions. Plasmids containing RhoA T19 and RhoA G14V were transfected. These constructs were obtained from the Guthrie cDNA Resource Center http://www.cdna.org. As control, parallel cells were transfected with empty pcDNA3.1+ plasmid (mock-transfected). After transfection for 24 h, cells (60-70% confluent) were treated with VEGF-C (100 ng/mL) for 48 h and cellular extracts were prepared according to the experiments to be performed.

Moesin on-target plus siRNA (J-011772-06), ROCK-2 siGENOME SMARTpool siRNA (L-004610-00) and scrambled control siRNA (D-001810-01) were obtained from Dharmacon. SiHa cells (40% confluent) were serum-starved for 1 h followed by incubation with 100 nM target siRNA or control siRNA (scrambled siRNA) for 6 h in serum-free media. The serum-containing media was then added (10% serum final concentration) for 42 h before experiments and/or functional assays were conducted. Target protein silencing was assessed through protein analysis up to 48 h after transfection.

### Cell migration assays

Cell migration was assayed with razor scrape assays as we previously described [14]. Briefly, a razor blade was pressed through the confluent SiHa cervical cancer cell monolayer into the plastic plate to mark the starting line. Cells were swept away on one side of that line. Cells were washed, and 2.0 mL of RPMI 1640 containing steroid-deprived FBS and gelatin (1 mg/mL) were added. Migration was monitored for 48 hours. Fresh medium and treatment were replaced every 12 h. Cells were digitally imaged and migration distance was measured by phase-contrast microscopy.

### Cell invasion assays

As we previously described [14], cell invasion were assayed following the standard method by using the BD BioCoat™ Growth Factor Reduced (GFR) Matrigel™ Invasion Chamber (BD Bioscience, USA). In brief, after rehydrating the GFR Matrigel inserts, the test substance was added to the wells. An equal number of Control Inserts (no GFR Matrigel coating) were prepared as control. 0.5 mL of SiHa cell suspension (2.5 × 10^4 ^cells/mL) was added to the inside of the inserts. The chambers were incubated for 48 h at 37°C, 5% CO_2 _atmosphere. After incubation, the non-invading cells were removed from the upper surface of the membrane using cotton tipped swabs. Then the cells on the lower surface of the membrane were stained with Diff-Quick stain. The invading cells were observed under the microscope at 100 × magnification. Cells were counted in the central field of triplicate membranes. The invasion index was calculated as the % invasion test cell/% invasion control cell.

### Patients, specimens, and immunohistochemical staining

Tissues were from cervical cancer patients hospitalized in the first hospital of Sun Yat-sen University (Guangzhou, China) with informed patient consent, including 24 patients with cervical intra-epithelial neoplasia (CIN), 22 patients with squamous carcinoma at stage I, and 20 patients with squamous carcinoma at stage II without radiotherapy or chemotherapy. The histological diagnosis was made according to the International Federation of Gynecology and Obstetrics (FIGO) staging system. Lymph node status was evaluated. Tissues from the normal cervix of 20 patients suffering from hysteromyoma who were undergone hysterectomy were taken as the control. All tissues were stored at -80°C until use.

Immunohistochemical staining was performed as we previously described [18]. Histological sections of 4 μm were mounted on silanized slides and allowed to dry for 1 h at room temperature (RT), followed by 1 h incubation in an oven at 60°C. Briefly, after deparaffination and rehydration, epitope retrieval was performed by immersing slides in DAKO Epitope Retrival Solution (0.01 M citrate buffer, pH 6.0) in a water bath at 98°C for 40 minutes followed by a 20 minutes cool-down period at room temperature (RT). The working dilution for the anti-human moesin monoclonal antibody (clone 38, Transduction Laboratories, Lexington, KY) was 1:50. The sections were incubated with primary antibodies at RT for 1 hour. Expression of moesin was evaluated according to the ratio of positive cells per specimen and staining intensity as described previously [19]. The ratio of positive cells per specimen was evaluated quantitatively and scored 0 for staining of ≤ 1%, 1 for staining of 2 to 25%, 2 for staining of 26 to 50%, 3 for staining of 51 to 75%, and 4 for staining ≥ 75% of the cells examined. Intensity was graded as follows: 0, no signal; 1, weak; 2, moderate; and 3, strong staining. A total score of 0 to 12 was finally calculated and graded as negative (-; score: 0-1), weak (+; 2-4), moderate (++; 5-8), and strong (+++; 9-12).

### Statistical analysis

Values are expressed as mean ± SD. Statistical differences between mean values were determined by ANOVA, followed by the Fisher's protected least significance difference (PLSD). To compare the differences of groups for immunohistochemistry, Kruskal Wallis Test and Chi-Square Test were used to calculate the P value. All differences were considered significant at P < 0.05.

## Results

### VEGF-C promoted SiHa cell migration and invasion and provoked actin cytoskeleton remodeling

**Figure 1:**
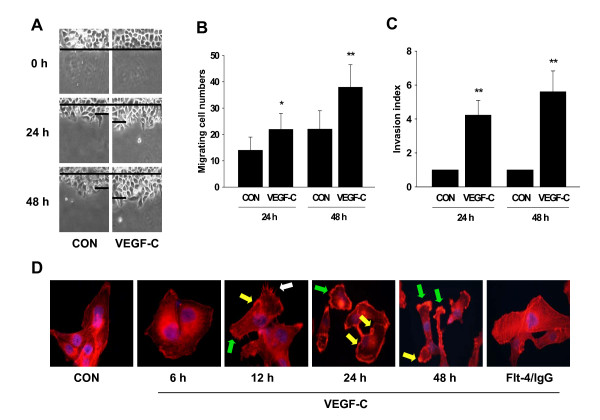
**VEGF-C increased SiHa cell migration and invasion and induced actin cytoskeleton remodeling**. (A) Cells were treated with VEGF-C (100 ng/mL) for 24 or 48 h and cell migration was assayed. SiHa cells were scraped out of the cell culture dish and the extent of migration of the remaining cells was assayed. The upper black lines indicate the starting line and the lower black lines indicate the mean migration distance. (B) Migrating cell numbers were measured and data representing the migration cell numbers from the starting line are expressed as mean ± SD. * = P < 0.05 vs. 24 h control; ** = P < 0.01 vs. 48 h control. (C) Cells were treated with VEGF-C (100 ng/mL) for 24 or 48 h. Cell invasion was assayed using invasion chambers. Invading cells were counted in three different central fields of triplicate membranes and invasion indexes are shown. ** = P < 0.01 vs. control. (D) SiHa cells were treated with VEGF-C (100 ng/mL) for 6 h, 12 h, 24 h or 48 h alone, or treated with VEGF-C for 48 h in the presence of Flt-4/IgG (100 ng/mL). Immunofluorescent analysis of Texas Red-phalloidin (in red) revealed the spatial modifications of actin fibres and the formation of specialized cell membrane structures. Green, white and yellow arrows indicate lamellipodia, pseudopodia and focal adhesion complexes, respectively. Nuclei were counterstained in blue. All the experiments were repeated three times with consistent results, and a representative result is shown.

First we observed the direct impact of VEGF-C on SiHa cell migration and invasion. As shown in Fig. 1A-B, the stimulation with VEGF-C (100 ng/mL) for 24 or 48 h showed increased number of cells migrating across the starting line, as compared to the control. Consistently, the number of cells that invaded the matrix with the treatment of VEGF-C (100 ng/mL) for 24 or 48 h in the inserts with GFR Matrigel was much higher than that in the control, as indicated by the value of invasion indexes (Fig. 1C).

It is well known that actin cytoskeleton remodeling is the initial step for cell migration and invasion. Therefore, we studied the effect of VEGF-C on actin rearrangement. At baseline, actin fibers in SiHa cell were arranged longitudinally in the cytoplasm and the cell membrane was regular (Fig. 1D). Treatment with VEGF-C (100 ng/mL) up to 12 h led to the shift of the actin fibers toward the edge of the membrane, in association with the formation of specialized membrane structures like filopodia, pseudopodia and focal adhesion complexes (Fig. 1D). These changes on actin organization could be observed in a majority of SiHa cells (about 75% of total cells). Flt-4/IgG is the fusion protein of human IgG with the extracellular ligand-binding domains of Flt-4. It competitively binds available VEGF ligands and, therefore, blocks respective VEGF activity by interfering with ligand-receptor interactions [20]. The Flt-4/IgG (100 ng/mL) abolished VEGF-C-induced actin rearrangement (Fig. 1D) in our study, suggesting that VEGF-C/Flt4 axis played a central role in these events.

### VEGF-C led to activation of the actin-regulatory protein, moesin

**Figure 2:**
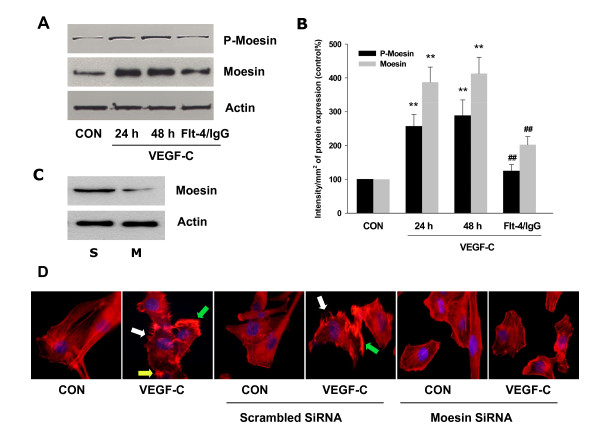
**VEGF-C led to activation of the actin-regulatory protein, moesin**. (A-B) SiHa cells were treated with VEGF-C (100 ng/mL) for 24 or 48 h alone, or treated with VEGF-C for 48 h in the presence of Flt-4/IgG (100 ng/mL). Total cell amount of wild-type (Moesin) or Thr^558^-phosphorylated moesin (P-Moesin) or β-actin (Actin) are shown with western blot analysis. Densitometry values were adjusted to β-actin intensity and then normalized to expression from the control sample. ** = P < 0.01 vs. corresponding control. ## = P < 0.01 vs VEGF-C 48 h. (C) SiHa cells were transfected with scrambled siRNA (-S) or moesin targeted siRNA (-M) for 48 h. After that the level of moesin expression was detected by western blot as indicated. β-actin was used as the loading control. (D) SiHa cells were treated with VEGF-C (100 ng/mL) for 48 h, in the presence or absence of scrambled siRNA or Moesin siRNA. Cells were stained and analyzed by immunofluorescence. Green, white and yellow arrows indicate lamellipodia, pseudopodia and focal adhesion complexes, respectively. All the experiments were repeated three times with consistent results, and a representative result is shown.

Next we investigated the effect of VEGF-C on the expression and phosphorylation of moesin, the protein that belongs to ERM (ezrin/radixin/moesin) family and regulates actin fibers rearrangement. Indeed, VEGF-C (100 ng/mL) significantly enhanced moesin expression as well as phosphorylation (Fig. 2A, B), which was blocked by Flt-4/IgG (Fig. 2A, B).

To confirm the role of moesin in VEGF-C-induced actin cytoskeleton remodeling, we silenced moesin by using specific siRNA (Fig. 2C: S - Scrambled SiRNA; M - Moesin SiRNA). As shown in Fig. 2D, the transfection with moesin siRNA eliminated VEGF-C effect on actin fibers rearrangement.

### VEGF-C activated moesin through RhoA/ROCK-2 pathway

**Figure 3:**
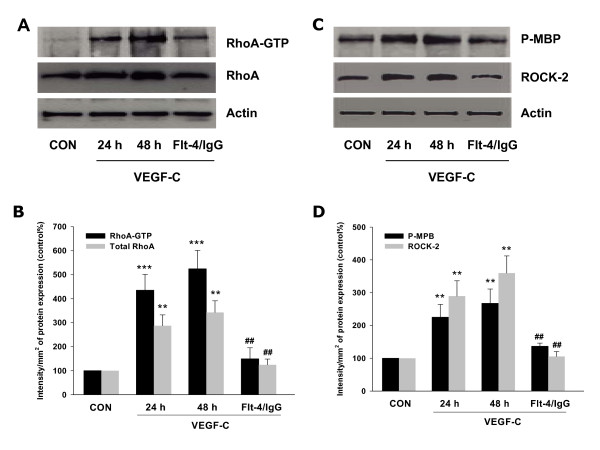
**VEGF-C activated RhoA/ROCK-2 pathway**. (A-B) RhoA expression and its activity were assayed in cells treated with VEGF-C (100 ng/mL) for 24 or 48 h alone, or treated with VEGF-C for 48 h in the presence of Flt-4/IgG (100 ng/mL). Active, GTP-bound RhoA was immunoprecipitated with Rhoteckin and subsequently assayed with western analysis with an anti-RhoA Ab. ** = P < 0.01 vs. corresponding control; *** = P < 0.001 vs. corresponding control; ## = P < 0.01 vs VEGF-C 48 h. (C-D) ROCK-2 expression and its activity were assayed in cells treated as indicated. ROCK-2 was immunoprecipitated with a specific Ab and the IPs were used to phosphorylate the bait protein, myelin basic protein (MBP). ROCK-2 kinase activity is shown as the amount of phosphorylated MBP (P-MBP). ** = P < 0.01 vs. corresponding control; ## = P < 0.01 vs VEGF-C 48 h. All the above experiments were performed in triplicates and representative images are shown.

**Figure 4:**
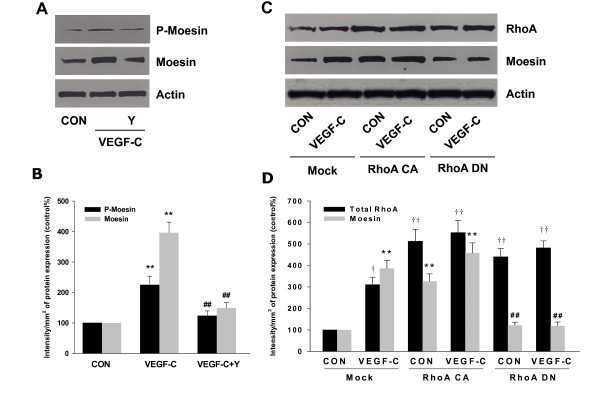
**VEGF-C activated moesin through RhoA/ROCK-2 pathway**. (A-B) SiHa cells were treated with VEGF-C (100 ng/mL) for 48 h in the presence or absence of the ROCK-2 inhibitor, Y-27632 (Y - 10 μM) and moesin and phosphorylated moesin were assayed with western analysis. β-actin was used as the loading control. ** = P < 0.01 vs. corresponding control; ## = P < 0.01 vs VEGF-C 48 h. (C-D) SiHa cells were either mock-transfected (-Mock) or exposed to constitutively active or dominant-negative RhoA (- RhoA CA or - RhoA DN). Cells were then treated with VEGF-C (100 ng/mL) for 48 h and wild type and P-moesin were analyzed. ** = P < 0.01 vs. mock-transfected control; ## = P < 0.01 vs VEGF-C in mock or RhoA CA transfected group, or RhoA CA transfected alone. † = P < 0.05 vs. total RhoA in mock control; †† = P < 0.01 vs. total RhoA in mock control. All the above experiments were performed in triplicates and representative images are shown.

**Figure 5:**
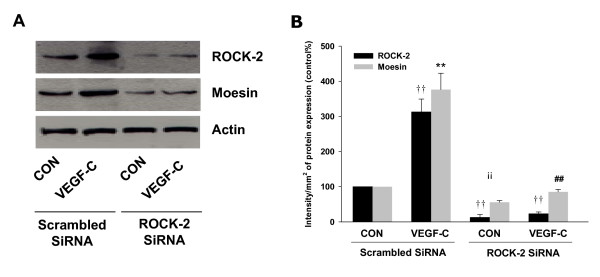
**ROCK-2 silencing blocked VEGF-C effect on moesin activation**. (A-B) Cells were exposed to VEGF-C (100 ng/mL) after transfection with 100 nM target siRNA for ROCK-2 or scrambled siRNA for 48 h. Cell contents of wild-type or phosphorylated moesin are shown ** = P < 0.01 vs. corresponding scrambled siRNA control; ## = P < 0.01 vs VEGF-C in scrambled siRNA group; †† = P < 0.01 vs. ROCK-2 in scrambled siRNA control. The experiment was performed in triplicates and representative images are shown.

Previously we have demonstrated that RhoA/ROCK-2 cascade is the upstream signaling of moesin activation [14-16,21]. In line with those findings, here we found that VEGF-C markedly enhanced total protein expression of RhoA/ROCK-2 and promoted their activities in SiHa cells (Fig. 3A-D). These effects were inhibited by Flt-4/IgG (Fig. 3A-D). Furthermore, blockade of ROCK-2 with specific inhibitor Y-27632 largely impaired VEGF-C-induced moesin expression and phosphorylation (Fig. 4A, B), suggesting the importance of RhoA/ROCK-2 cascade in VEGF-C-induced moesin expression and activation. To further test hypothesis of the crucial role of RhoA in the signaling of VEGF-C/Flt4 axis, moesin expression was studied with transient transfection of a RhoA constitutively active construct (RhoA CA - RhoA G14V) and a dominant negative RhoA construct (RhoA DN - RhoA T19N). It showed that moesin expression was ligand-independently enhanced by Rho CA and markedly inhibited by Rho DN (Fig. 4C, D). In parallel, silencing of ROCK-2 with siRNAs prevented the VEGF-C-enhanced moesin expression (Fig. 5A, B). These results confirmed that VEGF-C/Flt4 axis activated moesin by the recruitment of RhoA/ROCK-2 pathway.

### RhoA/ROCK-2/moesin cascade was involved in VEGF-C-enhanced cell migration and invasion

**Figure 6:**
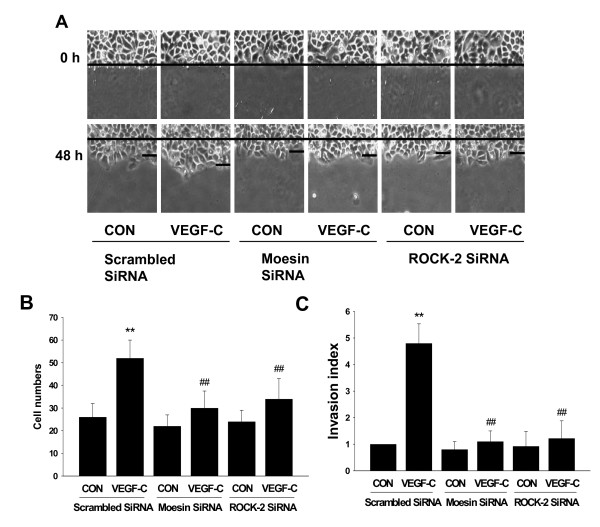
**RhoA/ROCK-2/moesin cascade was involved in VEGF-C-enhanced cell migration and invasion**. (A-B) Cells were treated with VEGF-C (100 ng/mL) for 48 h after transfection with scrambled siRNA, moesin siRNA or ROCK-2 siRNA. The upper black lines indicate the starting line and the lower black lines indicate the mean migration distance. Cell migration was assayed and the migration cell numbers from the starting line are expressed as mean ± SD. ** = P < 0.01 vs. scrambled siRNA control; ## = P < 0.01 vs. VEGF-C in scrambled siRNA group. (C) Cells were treated with VEGF-C (100 ng/mL) for 48 h after transfection with scrambled siRNA, moesin siRNA or ROCK-2 siRNA. Cell invasion was assayed using invasion chambers and invasion indexes were shown. The experiments were performed in triplicates. ** = P < 0.01 vs. scrambled siRNA control; ## = P < 0.01 vs. VEGF-C in scrambled siRNA group.

To determine the exact roles of moesin and RhoA/ROCK-2 signaling implicated in SiHa cell migration and invasion, we transfected siRNAs against moesin and ROCK-2 into cells. As expected, both siRNAs dramatically inhibited VEGF-C enhanced-cell migration and invasion (Fig. 6A, B, C).

### The expression levels of VEGF-C and moesin were positively correlated with tumor malignancy and metastasis

**Figure 7:**
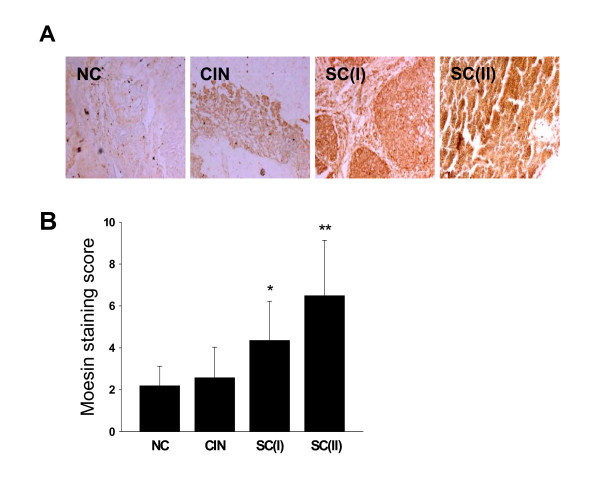
**The expression levels of VEGF-C and moesin were positively correlated with tumor malignancy**. (A) Immunohistochemical staining of moesin in normal cervical tissues and in cervical cancer at different stages. Normal cervical tissues (NC) and cervical intraepithelial neoplasia (CIN) showed weak moesin staining. In squamous carcinoma at stage I (SC(I)) or II (SC(II)), the moesin staining was significantly stronger than that of the NC and CIN samples. Original magnifications, × 40. (B) The average staining scores were shown. All the samples collected were immunohistochemical stained and representative images are shown.

To further examine whether the moesin expression is altered in cervical cancer, the expression pattern of moesin were studied in normal cervix and human cervical cancer tissues by immunohistochemistry. Moesin was found expressed predominantly in the cytoplasm of normal and cancer cells. The moesin staining in normal cervical samples (NC) and cervical intraepithelial neoplasia (CIN) samples was weak, which average staining score was 2.2 ± 0.92 and 2.6 ± 1.44, respectively (Fig. 7A, B). In squamous carcinoma at stage I (SC(I)) or II (SC(II)), the moesin staining was significantly stronger than that of the NC and CIN samples, which average score was 4.4 ± 1.86 and 6.5 ± 2.63, respectively (Fig. 7A, B).

To investigate if the moesin expression might be associated with the cervical cancer malignancy or metastasis, the moesin expression level and the clinicopathologic characteristics of 42 cervical squamous cancer patients were compared as summarized in Table 1. These results provided evidence that high expression of moesin was correlated with the malignant grade of cervical cancer (by Kruskal Wallis Test, X^2 ^= 22.46, P < 0.001) and with the presence of lymph node metastasis (by Chi-Square Test, X^2 ^= 15.78, P < 0.01).

**Table 1:** Clinicopathological associations of moesin expression in patients with cervical cancer

		Moesin immunostaining			
		
	Total number of cases	-	+	++	+++
Normal cervical tissues (**NC**)	20	8 (40%)	11 (55%)	1 (5%)	0 (0%)
cervical intraepithelial neoplasia (**CIN**)	24	2 (8.3%)	18 (75%)	3 (12.5%)	1 (4.2%)
Cervical squamous carcinoma	42	4 (9.5%)	13 (31%)	15 (35.7%)	10 (23.8%)
stage I (**SC(I)**)	22	2	8	8	4
stage II (**SC(II)**)	20	2	5	7	6
Lymph node metastasis					
With	10	0	1	2	7
Without	32	4	12	13	3

NOTE: Interpretation of moesin staining was described in Materials and Methods. Moesin staining was graded as negative (-; score: 0-1), weak (+; 2-4), moderate (++; 5-8), and strong (++; 9-12).

## Discussion

Cervical cancer is one of the most frequent types of tumor worldwide and its metastasis is the leading cause of death in patients with cervical cancer. However, the current understanding on the molecular mechanisms of cervical cancer metastasis is unclear. In this study, we demonstrated that VEGF-C accelerated cervical cancer metastasis by directly driving cancer cell migration and invasion. These processes were closely related to the effects of VEGF-C on moesin expression and activation through RhoA/ROCK-2 signaling pathway. To our knowledge, this is the first time reporting that moesin is the target protein of VEGF-C and responsible for cervical cancer metastasis.

Tumor metastasis is a complex process. A number of cellular alterations occur as cancer cell spread to lymph nodes and distant organs, including cellular transformation and tumor growth, angiogenesis and lymphangiogenesis. VEGF-C, the dimeric glycoprotein belonging to VEGF family of cytokines, plays critical role in a most of aggressive tumors. Indeed, the elevated level of serum VEGF-C has been found in patients with breast cancer [22], lung cancer [23] and cervical cancer [8] and it appears to be a unique marker for an early diagnosis of cancer metastasis. Moreover, increased VEGF-C mRNA expression in tumor tissues correlates positively with lymphatic metastasis and poor prognosis [24-26].

VEGF-C is mainly produced by tumor and stromal cells and it induces lymphangiogenesis that promotes the growth and metastasis of neoplasms. These biological effects are predominantly elicited by the activation of VEGF-C specific receptor Flt-4 [27,28]. It has been proposed that Flt-4 is restricted to be expressed on the lymphatic endothelium and tumor blood vessels [29]. However, recent studies have indicated that Flt-4 is also expressed in a variety of human malignancies [28], indicating that VEGF-C may affect cancer development and progression by direct effects on tumor cells. In agreement, we found that VEGF-C/Flt-4 axis drove cervical cancer cell horizontal migration and three-dimensional invasion into matrices, which are consistent with limited reports that VEGF-C directly stimulated the motility of other types of cancer cells [10,30,31], confirming that in addition to the regulatory actions on lymphangiogenesis, VEGF-C can promote tumor cell metastasis by directly triggering cell migration and invasion in an autocrine fashion.

While a lot is known on the molecular mechanisms of VEGF-C on lymphangiogenesis [32], little information is available on VEGF-C's direct impact on tumor cell motility. Reorganization of the actin cytoskeleton is the primary mechanism of cell motility and is essential for most types of cell migration [33]. For example, our previous studies have demonstrated that actin cytoskeleton remodelling is the initial process for breast cancer metastasis [14,34,35]. In this work, we indicated that VEGF-C provoked actin fibers rearrangement in cervical cancer cells and increased the formation of specialized membrane structures, which may interact with the extra-cellular matrix and with nearby cells, thus allowing the tumor cells to achieve locomotion. In analogy to our study, it's also reported by other groups that VEGF or VEGF-C induced actin reorganization and cell shape change in vascular endothelial cells, leading to the sprouts of endothelial cells [36,37].

Moesin, a member of the ERM family, is an actin-binding protein that plays a imperial role in cell motility by linking the actin cytoskeleton to a variety of membrane-anchoring proteins [38,39]. When activated through phosphorylation of Thr^558^, moesin induces actin de-polymerization and re-assembly toward the cell membrane edge, being responsible for the formation of cortical actin complexes. Our data showed that VEGF-C up-regulated moesin expression and phosphorylation and the silencing of moesin abolished VEGF-C's impact on actin rearrangement, indicating that moesin is the target protein for VEGF-C and the key mediator for actin cytoskeleton remodelling. This may be strong evidence to understand the mechanism that high moesin expression correlates with lymph node metastasis. Actually, high expression of ERM proteins has been identified as the prognostic markers in clinical cancers [40] and the expression pattern of moesin can be regarded as an independent prognostic factor in patients with oral squamous cell carcinoma [41]. Therefore, moesin expression may serve as a potential marker for cervical cancer metastasis, which need be further clinically investigated.

As previously reported [21,42,43], RhoA is a crucial mediator conveying the upstream signaling evoked by various factors to its downstream target ROCK-2, which is a known activator of ERM proteins. In this study, we showed that VEGF-C increased RhoA/ROCK-2 expression and activities. Moreover, the use of RhoA dominant negative construct or the inhibitor of ROCK-2 blocked VEGF-C-enhanced moesin expression and phosphorylation, implying that RhoA/ROCK-2 represents the link between VEGF-C and the activation of moesin. Indeed, the significance of RhoA/ROCK-2 in VEGF signalling has been displayed in endothelial cells, where RhoA/ROCK-2 mediates VEGF functions on microvascular permeability [44], endothelial migration and angiogenesis [45]. More importantly, the overexpression of RhoA is often observed in clinical cancers [46] and it has been repeatedly identified as a gene associated with metastasis [47,48]. These findings may partly be elucidated by our data that RhoA/ROCK-2 was able to activate moesin and finally led to enhanced cell motility. However, it should be pointed out that our data is mainly obtained from *in vitro *experiments. Whether the linkage between VEGF-C, RhoA/ROCK-2 and moesin exists and functions *in vivo *as we expected remains obscure. Our future studies will be aimed to further define this issue and one possible way is to suppress these proteins expression respectively with effective approaches, such as siRNA gene therapy in cervical cancer mice model and then their relationship and functions in cancer metastasis will be elucidated.

VEGF-C-enhanced migration and invasion of SiHa cell is markedly inhibited by moesin or ROCK-2 specific siRNAs, suggesting that RhoA/ROCK-2/moesin cascade plays an important role in these processes. Notwithstanding, this is not the exclusive way that VEGF-C promotes cancer cell motility. For example, it has been shown that VEGF or VEGF-C promoted cancer cell metastasis by up-regulation of integrin αvβ6 expression or of the neural cell adhesion molecule contactin-1 through divergent cellular signalings [10,31]. Moreover, other members of ERM family, such as ezrin, function similarly as moesin in cancer cells. Therefore, further efforts should focus on the exact function of these proteins involving cervical cancer metastasis.

## Conclusion

Taken together, we demonstrated that VEGF-C activated RhoA/ROCK-2/moesin cascade in cervical cancer cells, leading to the accelerated cancer cell migration and invasion. Genes and proteins within this signaling pathway may be the potential targets to develop therapeutic strategy for patients suffering from advanced cervical cancer.

## Abbreviations

VEGF-C: Vascular endothelial growth factor C; ROCK: Rho-associated kinase; ERM: ezrin/radixin/moesin.

## Competing interests

The authors declare that they have no competing interests.

## Authors' contributions

MH designed and carried out the experiments, analyzed the data, drafted and revised the manuscript; YC, WL, QSL, JXL and JHH carried out the experiments; XDF designed the experiments, analyzed the data, drafted and revised the manuscript. All authors read and approved the final manuscript.

## Pre-publication history

The pre-publication history for this paper can be accessed here:

http://www.biomedcentral.com/1471-2407/10/170/prepub
